# Exploring the Therapeutic Potential of 5-Fluorouracil-Loaded Calcium Carbonate Nanoparticles Combined with Natural Compound Thymoquinone for Colon Cancer Treatment

**DOI:** 10.3390/pharmaceutics16081011

**Published:** 2024-07-30

**Authors:** Xi Deng, Zhongming Yang, Kim Wei Chan, Md Zuki Abu Bakar

**Affiliations:** 1 Natural Medicines and Products Research Laboratory, Institute of Bioscience, Universiti Putra Malaysia, Serdang 43400, Selangor, Malaysia; dengxi9528@gmail.com (X.D.); yzm719268164@gmail.com (Z.Y.); chankim@upm.edu.my (K.W.C.); 2 Department of Veterinary Preclinical Science, Faculty of Veterinary Medicine, Universiti Putra Malaysia, Serdang 43400, Selangor, Malaysia

**Keywords:** therapeutic potential, 5-fluorouracil-loaded calcium carbonate nanoparticles, thymoquinone, combination therapy, colon cancer

## Abstract

Given the need for novel and effective therapies for colon cancer, this study aimed to investigate the effects of 5-fluorouracil-loaded calcium carbonate nanoparticles (5FU-CaCO_3_np) combined with thymoquinone (TQ) against colon cancer. A shaking incubator and a high-speed homogenizer were used to prepare the optimal 5FU-CaCO_3_np, with characterizations of physicochemical properties, in vitro drug release profile, and biocompatibility. In vitro experiments and molecular docking were employed to evaluate the therapeutic potential of the combination for colon cancer treatment. Study results revealed that 5FU-CaCO_3_np with a size of approximately 130 nm was synthesized using the high-speed homogenizer. Its favorable biocompatibility, pH sensitivity, and sustained release properties facilitated reduced toxic side effects of 5-FU on NIH3T3 normal cells and enhanced inhibitory effects on CT26 colon cancer cells. The combination of 5FU-CaCO_3_np (1.875 μM) and TQ (30 μM) showed significantly superior anti-colon cancer effects to 5FU-CaCO_3_np alone in terms of cell proliferation and migration inhibition, cell apoptosis induction, and spheroid growth suppression in CT26 cells (*p* < 0.05), with strong interactions between the drugs and targets (E-cadherin, Bcl-2, PCNA, and MMP-2). These results provide evidence for 5FU-CaCO_3_np as a novel regimen against colon cancer. Combining 5FU-CaCO_3_np and TQ may offer a new perspective for colon cancer therapy.

## 1. Introduction

Cancer is a major public health issue around the world, with the World Health Organization reporting nearly 10 million cancer-related deaths in 2022 [1]. Among various cancers, colon cancer ranks third in incidence and second in mortality, remaining a significant threat to global health. Chemotherapy drug 5-fluorouracil (5-FU) has been widely used to treat various cancers since 1957 and is the first-line therapy for colon cancer treatment [2,3]. As an anti-metabolite drug, 5-FU can inhibit thymidylate synthase and incorporate its metabolites into RNA and DNA against cancers [4,5]. However, the disadvantages of 5-FU, such as nonspecific targeting, poor solubility, dose-dependent toxicity on normal tissues, and minimal tumor accumulation, greatly limit its clinical application [6,7].

Nanotechnology advancements have made nanoparticle-based drug delivery systems a promising candidate for effective cancer treatment [8,9,10]. Many nanoparticle-based drug delivery systems have been applied to deliver 5-FU for cancer treatment to enhance the anticancer efficacy of 5-FU and decrease its toxic side effects. For example, 5-FU-loaded lactoferrin nanoparticles were synthesized for malignant melanoma, whereas 5-FU was loaded into the mesoporous silica-based hybrid nanoparticles to enhance efficacy against colon cancer [11,12]. Calcium carbonate (CaCO_3_) is a polymorphic material abundant in nature, widely existing in shells, animal bones, and stalactites, where aragonite is a stable phase of CaCO_3_ [13]. Moreover, CaCO_3_ has excellent pH sensitivity, biodegradability, and biocompatibility, which makes CaCO_3_ nanoparticles (CaCO_3_np) one of the active research fields in cancer treatment [14,15].

Thymoquinone (TQ) is a phytochemical compound isolated from black cumin (*Nigella sativa*) and used as an anti-inflammatory, analgesic, and antioxidant agent in traditional medicine [16,17]. Since a study in 2003 noted that TQ could inhibit growth and induce apoptosis of cancer cell lines, TQ has attracted more and more attention in cancer treatment [18]. The anti-colon cancer effects of TQ have been proven in previous reports [17,19]. Moreover, combining chemotherapeutic drugs and natural compounds has become a novel multi-targeted approach to cancer treatment, enhancing the effectiveness of chemotherapy drugs and mitigating their toxic side effects [20]. Kensara et al. [21] demonstrated that 5-FU combined with TQ was more effective than the individual agents in colorectal cancer rats, with a synergistic effect twice that of the individual therapies. However, no literature has investigated the combinatorial use of 5-FU-loaded nanoparticles and TQ for cancer treatment.

As a result of these premises, the purpose of the present study was to explore the therapeutic potential of 5-FU-loaded CaCO_3_np (5FU-CaCO_3_np) combined with TQ for colon cancer. The optimal formulation of 5FU-CaCO_3_np was obtained by comparing the loading results of a shaking incubator and a high-speed homogenizer and the results of different weight ratios between 5-FU and CaCO_3_np. A series of in vitro experiments were designed to investigate the effects of 5FU-CaCO_3_np in combination with TQ on colon cancer cell proliferation, apoptosis, migration, and spheroid growth. Moreover, these effects were validated by the molecular docking approach at a molecular level.

## 2. Materials and Methods

### 2.1. Materials

Cockle shells were sourced from the local market in Serdang, Malaysia; dodecyl dimethyl betaine (BS-12, Jindun Industrial, Shanghai, China); 5-fluorouracil (5-FU, Molekula, Darlington, UK); deionized water with a resistance of 18.2 MΩ.cm was obtained from an ELGA LabWater PURELAB flex3 water purification system (Type I) (ELGA, High Wycombe, UK); brine shrimp (*Artemia salina*) was purchased from a local pet shop in Kuala Lumpur, Malaysia; NIH3T3 embryonic fibroblast and CT26 colon cancer cell lines originated from the American Type Culture Collection (ATCC, Manassas, VA, USA); RPMI-1640 with L-glutamine (Cytiva, Freiburg, Germany); fetal bovine serum (FBS, Cytiva, Freiburg, Germany); penicillin–streptomycin (Elabscience, Wuhan, China); trypsin-EDTA (1×) (Cytiva, Freiburg, Germany); 3-(4,5-dimethylthiazol-2-yl)-2,5-diphenyltetrazolium bromide (MTT, Solarbio, Beijing, China); dimethylsulfoxide (DMSO, ChemAR, Kielce, Poland); thymoquinone (TQ, Molekula, Darlington, UK); crystal violet (Solarbio, Beijing, China); acridine orange (AO, Sigma-Aldrich, St. Louis, MO, USA); propidium iodide (PI) and ribonuclease A (RNase A) (Nacalai Tesque, Kyoto, Japan); Annexin V-FITC apoptosis kit (BD Bioscience, Fremont, CA, USA). All other reagents were of analytical grade.

### 2.2. Preparation of CaCO_3_np from Cockle Shells

Micro-sized calcium carbonate from cockle shells was prepared initially. Briefly, cockle shells were boiled for 30 min in a steel container and thoroughly washed to remove stains on the shells. Upon drying in an oven (FD 115, Fisher Scientific, Limburg, Germany) at 50 °C, cockle shells were finely ground and sieved using a stainless-steel laboratory test sieve (Endicott Ltd., London, UK) with an aperture size of 75 μm to obtain micro-sized calcium carbonate powder. Subsequently, 5 g of micro-sized calcium carbonate powder was added into 50 mL of deionized water in a flat-bottom flask and stirred with a magnetic stirrer bar at 1000 rpm for 30 min at room temperature using a magnetic stirring machine (WiseStir^®^ Systematic Multi-Hotplate Stirrer, Daihan Scientific^®^, Wonju, Republic of Korea). Following this, 1.5 mL of BS-12 was added and stirred at 1000 rpm for 2 h at room temperature. The obtained samples were then centrifuged (Beckman Coulter, Brea, CA, USA) three times to remove BS-12 and dried in an oven at 50 °C. The dried samples were placed in a glass cylindrical jar containing ceramic balls rolling on a programmable ball miller (BML-6″, Daihan Scientific^®^, Wonju, Republic of Korea) at 200 rpm for 500 h to obtain the final CaCO_3_np.

### 2.3. Synthesis and Optimization of 5FU-CaCO_3_np

5FU-CaCO_3_np was synthesized using both a shaking incubator and a high-speed homogenizer with different weight ratios of 5-FU to CaCO_3_np (1:2, 1:4, 1:6, 1:8, 1:10, 1:15, and 1:20 *w*/*w*). When the shaking incubator (LSI 3016R, Daihan Labtech, Wonju, Republic of Korea) was employed for 5-FU loading, the mixtures (12 mg of CaCO_3_np and varying amounts of 5-FU in 1.2 mL of deionized water) were continuously shaken at 200 rpm overnight in the dark at room temperature. When the high-speed homogenizer (IKA-T25 digital Ultra-Turrax, Staufen, Germany) was employed, the mixtures (150 mg of CaCO_3_np and varying amounts of 5-FU in 15 mL of deionized water) were homogenized at the time and speed determined by single-factor experiments (the single-factor experimental design is shown in Table 1). Consequently, 5FU-CaCO_3_np was collected after centrifuging, washing, and drying and kept at room temperature for further use.

**Figure 1:**
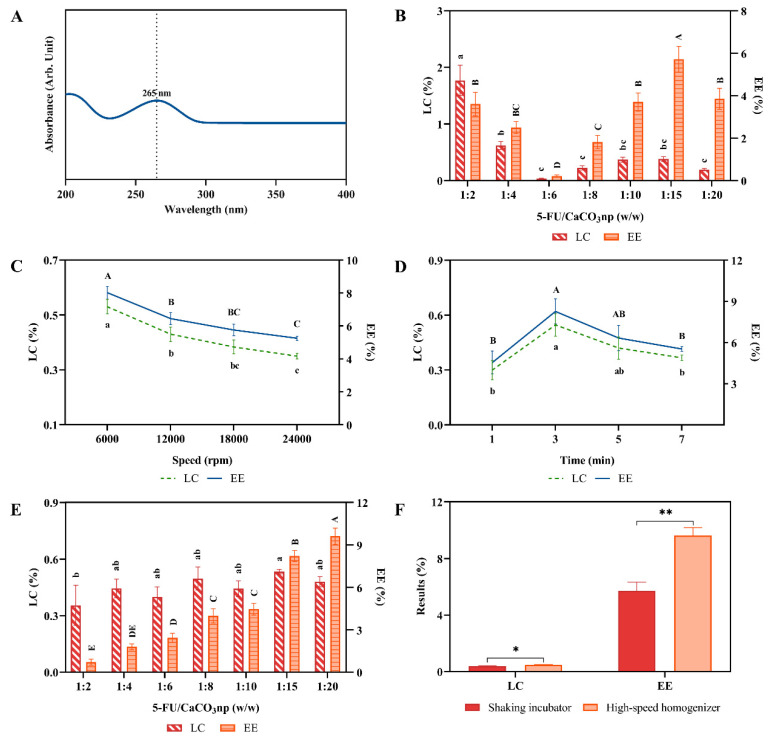
(**A**) Absorption spectrum of 5-FU with the absorbance peak (265 nm) in deionized water; (**B**) LC and EE of 5FU-CaCO_3_np at different weight ratios of 5-FU to CaCO_3_np when using the shaking incubator for 5-FU loading; (**C**) LC and EE of 5FU-CaCO_3_np at different speeds in the single-factor experiment; (**D**) LC and EE of 5FU-CaCO_3_np at different times in the single-factor experiment; (**E**) LC and EE of 5FU-CaCO_3_np at different weight ratios of 5-FU to CaCO_3_np when using the high-speed homogenizer for 5-FU loading; (**F**) comparison of the optimal loading results obtained using the shaking incubator and high-speed homogenizer. The results with the different superscripts (a–c and A–E nomenclature) indicate significant differences (*p* < 0.05). * *p* < 0.05, ** *p* < 0.01. LC, loading content; EE, encapsulation efficiency.

The supernatant of every formulation was collected for drug loading content (LC) and encapsulation efficiency (EE) analyses. The superior synthesis method and the best formulation were chosen by comparing the results of LC and EE. The weight of the free drug in the supernatant was determined using an Ultraviolet–visible (UV-Vis) spectrophotometer (PerkinElmer Lambda 35, Perkin Elmer, Waltham, MA, USA), where the absorption peak of 5-FU in deionized water was 265 nm (Figure 1A). The results of LC and EE were calculated using the following equations:

LC (%) = (weight of drug fed − weight of free drug)/weight of nanoparticles × 100%,

EE (%) = (weight of drug fed − weight of free drug)/weight of drug fed × 100%.

### 2.4. Characterization of 5FU-CaCO_3_np

#### 2.4.1. Morphology, Size, and Surface Charge

The morphology of 5FU-CaCO_3_np was analyzed by field-emission scanning electron microscopy (FESEM, Nova^TM^NanoSEM 230, FEI, Hillsboro, OR, USA) and high-resolution transmission electron microscopy (HRTEM, Hitachi H-7100, Tokyo, Japan). Briefly, 5FU-CaCO_3_np was suspended in 100% acetone and then sonicated. Subsequently, a drop of the dispersion was put on a carbon-coated copper grid, and the sample was dried at room temperature before the microscopy viewing. The particle size in the HRTEM figure was measured using ImageJ 1.51n software. The hydrodynamic size and surface charge of 5FU-CaCO_3_np were analyzed using a Zetasizer (Zetasizer Nano ZS, Malvern Instruments, Malvern, UK). Briefly, 5FU-CaCO_3_np was dispersed in 100% acetone for hydrodynamic size analysis and in deionized water for surface charge analysis.

#### 2.4.2. Crystalline Nature and Chemical Property

The crystalline nature of 5FU-CaCO_3_np was determined using a Shimadzu X-ray diffraction (XRD)-6000 powder diffractometer (Kyoto, Japan) on a continuous scanning process with Cu Kα (λ = 1.5406 Å) as the X-ray source. The XRD test result was analyzed using Jade 6 software. The crystallite size of 5FU-CaCO_3_np was calculated using Scherrer’s equation:

D = 0.9λ/B × cosθ,(1)

where D is crystallite size in angstroms, λ is X-ray wavelength (1.5406 Å), B is full width at half maximum of peak in radian unit, and θ is peak position in radian unit. The chemical property of 5FU-CaCO_3_np was evaluated using Fourier transform infrared spectroscopy (FTIR, Spectrum 100, Perkin Elmer, Shelton, CT, USA) in the range of 4000 to 400 cm^−1^ at a 2 cm^−1^ resolution and averaging 64 scans. The FTIR test result was analyzed using OMNIC 7.3 software.

#### 2.4.3. In Vitro Drug Release Study

The drug release profile of 5FU-CaCO_3_np was monitored in PBS buffer with 1% (*v*/*v*) Tween 80 at pH 7.4, 6.5, and 5.0 using the pre-treated dialysis tubing cellulose membrane (molecular weight cut-off: 14 kDa; Sigma-Aldrich). The dialysis was conducted in a shaking incubator at 100 rpm at 37 ± 0.5 °C. At predetermined time intervals, the release media were withdrawn and replaced with the same volume of fresh media. The weight of 5-FU released from 5FU-CaCO_3_np was determined by UV-vis spectrophotometry. The drug release results were fitted in various kinetic models, including zero-order, first-order, Higuchi, Hixson–Crowell, and Krosmeyer–Peppas models, where the R^2^ value was used to evaluate the best fit of the results to the kinetic models, and the n value from the Krosmeyer–Peppas model was applied to analyze the drug release mechanism.

#### 2.4.4. Hemocompatibility Analysis

Hemocompatibility analysis of 5FU-CaCO_3_np was conducted using plasma collected from a healthy male Sprague Dawley rat via cardiac puncture into a heparin anticoagulant tube. The housing conditions of the animal were meticulously controlled, with a temperature of 24 ± 2 °C, humidity of 45 ± 5%, and a 12 h light/12 h dark cycle being maintained. All procedures adhered to the guidelines and received approval from the Institute Animal Care and Use Committee of Universiti Putra Malaysia (approval no. UPM/IACUC/AUP-R065/2023).

The red blood cells (erythrocytes) were collected from plasma by centrifuging at 3000 rpm for hemocompatibility analysis. The erythrocytes were redispersed in the PBS to prepare a 4% solution. The dispersed blood was added to a 5FU-CaCO_3_np aliquot with different concentrations (62.5–4000 μg/mL, in deionized water) and incubated for 1 h at 37 °C. Subsequently, the mixed samples were centrifuged, and the supernatant was analyzed at 540 nm by a microplate reader. The PBS solution and deionized water were used as negative (0% hemolysis) and positive control (100% hemolysis), respectively. The hemolysis percentage was calculated with the following equation:

hemolysis (%) = [(Abs_sample_ − Abs_negative_)/(Abs_positive_ − Abs_negative_)] × 100%,

where Abs_sample_, Abs_negative_, and Abs_positive_ represent the absorbance of the test sample, negative control, and positive control, respectively.

#### 2.4.5. Toxicity Analysis in Brine Shrimp

The toxicity of 5FU-CaCO_3_np was evaluated by measuring the survival rate of brine shrimp treated with different concentrations of 5FU-CaCO_3_np. Briefly, 34 g of sea salt (without iodine) was dissolved in 1 L of deionized water as the simulated seawater, and 1 g of brine shrimp eggs was put into the seawater. The eggs were then incubated with constant air and a light source (12 h of light–dark cycle) at room temperature. Upon hatching, 10 active nauplii in 1 mL of seawater were transferred to each well of the 24-well plate. Different concentrations (0–1000 μg/mL) of 5FU-CaCO_3_np were added into the wells containing the nauplii, whereas KMnO_4_ dissolved in seawater with the same concentrations served as the positive control. The surviving and dead nauplii were observed using light microscopy (LM) and recorded at 12, 24, 36, and 48 h. The brine shrimp survival rate was calculated using the following formula:

survival rate (%) = alive brine shrimp/total brine shrimp × 100%.

#### 2.4.6. Cytotoxicity Analysis

Cytotoxicity of 5FU-CaCO_3_np was evaluated in the NIH3T3 embryonic fibroblast cell line. Briefly, NIH3T3 cells were cultured with different concentrations of 5-FU (0–480 μM) and 5FU-CaCO_3_np (5-FU: 0–480 μM) for 96 h. After 96 h of incubation, 20 μL of MTT solution (5 mg/mL) was added into each well and incubated at 37 °C (5% CO_2_ and 95% air) for 4 h. All solution in each well was then replaced with 150 μL of DMSO to dissolve the purple formazan crystals. The absorbance was measured at 570 nm with reference wavelength 630 nm using the multimode microplate reader (Synergy H1, BioTek, Winooski, VT, USA). The cell viability was determined relative to the absorbance in the control group. Half maximum inhibitory concentration (IC_50_) was calculated by nonlinear regression analysis in GraphPad Prism 8 software.

### 2.5. In Vitro Cell Experiments of 5FU-CaCO_3_np Combined with Thymoquinone

#### 2.5.1. 3-(4,5-Dimethylthiazol-2-yl)-2,5-diphenyltetrazolium Bromide Assay

Colon cancer CT26 cells (5 × 10^3^ cells/well) were seeded in 96-well plates for 24 h and treated with different concentrations of 5FU-CaCO_3_np (5-FU: 0–480 μM, in deionized water), TQ (0–60 μM, in ethanol), or their combination for 96 h. After treatment, MTT (5 mg/mL) reagent was added into each well for 4 h of incubation. The purple formazan crystals formed were solubilized with 150 μL of DMSO, and the absorbance (OD) at 570 nm (630 nm as the reference wavelength) was measured using the multimode microplate reader (Synergy H1, BioTek, Winooski, VT, USA). The cell viability was determined relative to the absorbance in the control group. IC_50_ was calculated by nonlinear regression analysis in GraphPad Prism 8 software.

#### 2.5.2. Synergism Analysis

The combination index (CI) was calculated using the CompuSyn 1.0 software established by Chou and Talalay, where CI > 1 indicates antagonism, CI = 1 indicates additive, and CI < 1 indicates synergism [22].

#### 2.5.3. Colony Formation Assay

After being cultured in 6-well plates for 48 h, CT26 cells were treated with 5FU-CaCO_3_np (5-FU: 1.875 μM, in deionized water), TQ (30 μM, in ethanol), and their combination. The cells were then allowed to form colonies for 7 d of incubation. Once the colonies were visible, they were then fixed with 100% methanol and stained with 0.5% crystal violet. The cells were photographed using LM. The number of colonies was calculated using ImageJ software.

#### 2.5.4. Acridine Orange/Propidium Iodide Staining

Following 96 h of treatment with 5FU-CaCO_3_np (5-FU: 1.875 μM, in deionized water) and TQ (30 μM, in ethanol) either alone or in combination, CT26 cells were washed with PBS and stained with 5 μg/mL AO/PI (1:1) dual stain for 10 min at room temperature. The cells were photographed using an inverted fluorescence microscope (Carl Zeiss Microscopy GmbH, Gottingen, Germany). Number of AO-viable cells was calculated using ImageJ software according to the presence or absence of non-viable cell staining (PI).

#### 2.5.5. Cell Apoptosis Analysis

CT26 cells were cultured in 6-well plates and incubated with 5FU-CaCO_3_np (5-FU: 1.875 μM, in deionized water) and TQ (30 μM, in ethanol) alone or in combination for 96 h. After collection and centrifugation, the cells were resuspended in 1× binding buffer and stained with Annexin V-FITC (5 μL) and PI (5 μL) in the dark for 20 min. Cell apoptosis was then measured by the BD FACSCanto II flow cytometer (BD Biosciences, Milpitas, CA, USA) and analyzed by BD FACSDiva 6.1.2 software.

#### 2.5.6. Wound Healing Assay

CT26 cells were seeded in 6-well plates and scraped with a 200 μL sterile pipette tip when the cells achieved 90% confluence. The scratched cells were then incubated with 5FU-CaCO_3_np (5-FU: 1.875 μM, in deionized water) and TQ (30 μM, in ethanol) alone or in combination for 48 h. Images were captured using an inverted fluorescence microscope (Carl Zeiss Microscopy GmbH, Gottingen, Germany) after 0, 24, and 48 h. The wound healing area was measured using ImageJ software.

#### 2.5.7. Three-Dimensional Spheroid Inhibition Assay

CT26 cells (15,000 cells/well) were seeded in agarose-coated 96-well plates and incubated for 4 d to form CT26 spheroids. Spheroids were cultured with 5FU-CaCO_3_np (5-FU: 1.875 μM, in deionized water) and TQ (30 μM, in ethanol) alone or in combination for 96 h and imaged for 14 d using LM. The diameter and volume of spheroids were determined using Image J software.

### 2.6. Molecular Docking

The 3D structures of 5-FU and TQ were obtained from the PubChem database (https://pubchem.ncbi.nlm.nih.gov/), and the structures of E-cadherin (PDB ID: 4ZTE), Bcl-2 (PDB ID: 6O0K), PCNA (PDB ID: 8GLA), and MMP-2 (PDB ID: 8H78) were downloaded from the PDB database (https://www.rcsb.org/). Discovery Studio Visualizer v19.1.0.18287 software was used to remove water molecules of proteins, predict the binding sites of protein pockets, and delete the original ligands. The processed proteins were imported into AutoDock Tools 1.5.6 software to add polar hydrogen and charge. The molecular docking was executed using AutoDock Vina 1.2.5 software. The results of molecular docking were visualized in Discovery Studio Visualizer v19.1.0.18287 software.

### 2.7. Statistical Analysis

Data analysis was performed with at least triplicates using the GraphPad Prism 8 and Origin 9.8.0.200 softwares. Statistical analysis involved one-way ANOVA followed by Tukey’s post hoc test for multiple comparisons or Student’s *t*-test for single comparison and two-way ANOVA followed by Dunnett’s post hoc test. A *p* value < 0.05 (*p* < 0.05) was considered statistically significant. Unless otherwise stated, the results are expressed as mean ± standard deviation.

## 3. Results and Discussion

### 3.1. Preparation of 5FU-CaCO_3_np

A shaking incubator and a high-speed homogenizer were employed for loading 5-FU into CaCO_3_np, and their loading results were compared to obtain the optimal 5FU-CaCO_3_np. Drug loading content and encapsulation efficiency are the major parameters used to evaluate drug loading results [23]. 5-FU loading with different weight ratios of 5-FU to CaCO_3_np was first performed using the shaking incubator to screen the best formulation based on the loading results. Figure 1B shows the highest EE (5.72%) of 5FU-CaCO_3_np at the weight ratio of 1:15 with 0.38% LC. Before using the high-speed homogenizer for 5-FU loading, single-factor experiments were conducted to determine the optimal speed and time, which are the factors affecting the loading results of using the high-speed homogenizer drug loading [24]. As a result, loading at 6000 rpm reached the highest LC and EE (Figure 1C). Although the LC and EE of loading for 3 min had no significant difference compared with the values for 5 min (Figure 1D), the more effective 3 min was chosen as the time for 5-FU loading. Therefore, when the high-speed homogenizer was used for 5-FU loading, 6000 rpm and 3 min were the optimal process parameters.

Based on that, 5-FU loading with different weight ratios of 5-FU to CaCO_3_np was performed. As the findings in Figure 1E,F illustrate, the EE of 5FU-CaCO_3_np obtained the highest value (9.61%) at the weight ratio of 1:20 with the LC of 0.48%, surpassing those achieved by using the shaking incubator (*p* < 0.05). This is likely because the shaking incubator employs a simple agitation technique, while the high-speed homogenizer utilizes intense mechanical forces, enhancing the loading efficiency [25]. The high-speed homogenizer accelerates interactions between drug molecules and nanoparticles through vigorous mechanical action, resulting in quicker and more effective compound loading than the gentler agitation of a shaking incubator. Additionally, high-speed homogenization ensures uniform dispersion of drug molecules and prevents nanoparticle aggregation, thereby promoting a consistent and reproducible loading process [26]. Consequently, the high-speed homogenizer operating at 6000 rpm for 3 min coupled with the 1:20 weight ratio of 5-FU to CaCO_3_np emerged as the optimal formulation for loading 5-FU into CaCO_3_np.

### 3.2. Physicochemical Characterization and In Vitro Drug Release Profile of 5FU-CaCO_3_np

**Figure 2:**
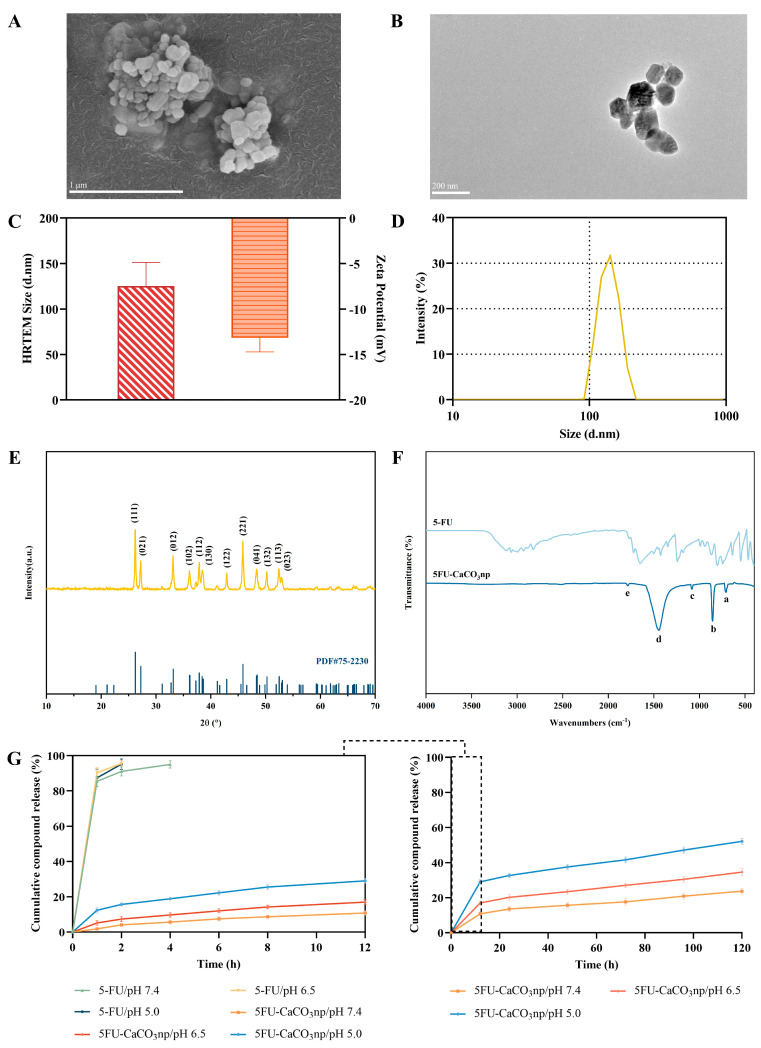
Surface morphology of 5FU-CaCO_3_np in (**A**) FESEM and (**B**) HRTEM; (**C**) size of 5FU-CaCO_3_np in HRTEM and surface charge; (**D**) hydrodynamic size of 5FU-CaCO_3_np; (**E**) XRD patterns of 5FU-CaCO_3_np and the reference diffraction peak (PDF#75-2230) of aragonite calcium carbonate nanoparticles from Inorganic Crystal Structure Database. The labels are the Miller indices planes (characteristic peaks of aragonite phase); (**F**) FTIR spectra of 5FU-CaCO_3_np and 5-FU. The labeled peaks (a–e) that represent peak assignments are stated in Table 2; (**G**) in vitro drug release profiles of 5FU-CaCO_3_np and 5-FU in PBS with 1% (*v*/*v*) Tween 80 at 37 °C and pH 7.4, 6.5, and 5.0. FESEM, field-emission scanning electron microscopy; HRTEM, high-resolution transmission electron microscopy; XRD, X-ray diffraction; FTIR, Fourier transform infrared spectroscopy; PBS, phosphate-buffered saline.

Figure 2A,B show that the FESEM and HRTEM images of 5FU-CaCO_3_np displayed homogeneous nearly-spherical-shaped porous nanoparticles. In addition, the HRTEM image revealed an average size (125 nm) of 5FU-CaCO_3_np (Figure 2C). Moreover, the surface charge and hydrodynamic size of 5FU-CaCO_3_np were analyzed by dynamic light scattering, with an average surface charge of −13.2 mV and an average hydrodynamic size of 133 nm (Figure 2D), consistent with the size in HRTEM. Nanoparticles sized between 20 and 150 nm effectively reduce liver clearance and kidney filtration, thereby prolonging their circulation time in vivo [27]. Furthermore, it has been reported that cationic nanoparticles are typically cleared from circulation more rapidly than anionic nanoparticles. In contrast, neutral nanoparticles and those with slightly negative charges exhibit extended circulating half-lives [28]. Therefore, the particle size and slightly negative surface charge of 5FU-CaCO_3_np are ideal for 5-FU delivery in colon cancer treatment.

In Figure 2E, it is shown that 5FU-CaCO_3_np with crystallinity more than 95% exhibited a purely aragonite structure as its characteristic diffraction peaks (crystal planes) observed, i.e., 26.22° (111), 27.23° (021), 33.15° (012), 36.12° (102), 37.90° (112), 38.42° (130), 42.92° (122), 45.87° (221), 48.33° (041), 50.25° (132), 52.48° (113), and 53.05° (023), conformed to the reference diffraction peaks (PDF#75-2230) of aragonite calcium carbonate nanoparticles from the Inorganic Crystal Structure Database. The crystallite size of 5FU-CaCO_3_np was 965 Å, confirming the size obtained from HRTEM and the Zetasizer. The FTIR spectra of 5-FU and 5FU-CaCO_3_np are displayed in Figure 2F, and the corresponding peak vibration assignments of 5FU-CaCO_3_np are presented in Table 2. The characteristic peaks of 5FU-CaCO_3_np at 710.68, 851.60, and 1082.21 cm^−1^ corresponded to in-plane C-O bending vibration, out-of-plane C-O bending vibration, and symmetric C-O stretching of CO_3_^2−^ in aragonite polymorph, respectively, indicating the aragonite crystal of 5FU-CaCO_3_np, consistent with the finding in the XRD test. Additionally, the prominent peak for the asymmetric C-O stretching of CO_3_^2−^ appeared at 1440.93 cm^−1^, and the weak peak at 1783.63 cm^−1^ was attributed to symmetric C-O stretching and in-plane C-O bending vibration of CO_3_^2−^. 5-FU had characteristic peaks at 544.13, 806.76, 1242.35, 1424.91, 1649.11, and 3119.2 cm^−1^ due to the vibration of imide stretch (amide II and amide III) and aromatic ring. The absence of characteristic peaks of 5-FU in the 5FU-CaCO_3_np spectra implies that no chemical reaction occurred between 5-FU and CaCO_3_np.

Figure 2G illustrates the release profiles of 5-FU from 5FU-CaCO_3_np in healthy tissue environments (pH 7.4), the extracellular environment of malignant tumors (pH 6.5), and the intracellular environment of malignant tumors (pH 5.0) [29]. It was revealed that the release of 5-FU from 5FU-CaCO_3_np was pH dependent. The accumulative 5-FU release amounts of 5FU-CaCO_3_np at pH 7.4 were 13.51% within 24 h and 23.57% within 120 h, while the release rates at pH 6.5 were higher than those at pH 7.4, with accumulative release amounts 20.19% within 24 h and 34.58% within 120 h. Nevertheless, 5-FU release rates at pH 5.0 accelerated more obviously, showing accumulative release amounts of 32.58% within 24 h and 52.12% within 120 h. Therefore, 5FU-CaCO_3_np exhibited pH sensitivity, potentially increasing the accumulation of 5-FU in cancer cells to enhance its anticancer efficacy.

Remarkably, there was a sudden release of 5-FU from 5FU-CaCO_3_np at pH 5.0 (15.64% within 2 h), which could be attributed to the physical adsorption of a small amount of 5-FU on the surface of CaCO_3_np, followed by a sustained release due to the slow diffusion. The initial sudden release of drugs swiftly achieves their effective therapeutic concentrations in the body, while sustained release maintains these drugs within the therapeutic concentration range [30]. In contrast, pure 5-FU showed a rapid release of more than 95% within 4 h at the three pH values. Compared to the fast release of pure 5-FU, the sustained release of 5FU-CaCO_3_np could maintain the effective concentrations of 5-FU, thus reducing its dose and adverse effects [31]. The release patterns of 5-FU from 5FU-CaCO_3_np at the three pH values were further analyzed using zero-order, first-order, Higuchi, Hixon–Crowell, and Korsmeyer–Peppas models. In Table 3, the Korsmeyer–Peppas model shows the best goodness of fit for 5FU-CaCO_3_np (R^2^ = 0.98258 at pH 7.4, R^2^ = 0.98988 at pH 6.5, and R^2^ = 0.99084 at pH 5.0), corresponding to the diffusion-controlled principal. Moreover, the release exponents (n values) revealed the Fickian diffusion mechanism of 5-FU released from 5FU-CaCO_3_np [32].

### 3.3. Biocompatibility of 5FU-CaCO_3_np

**Figure 3:**
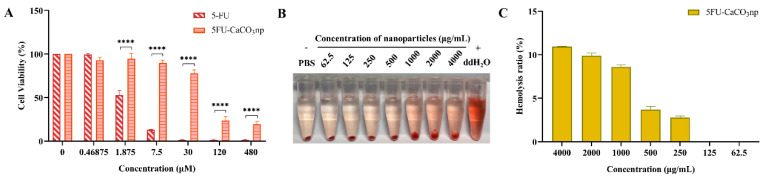
(**A**) Cytotoxicity of 5-FU and 5FU-CaCO_3_np (5-FU concentrations: 0–480 μM) in NIH3T3 embryonic fibroblast cell line for 96 h; (**B**) hemolysis phenomena and (**C**) hemolysis ratios of 5FU-CaCO_3_np. PBS solution and ddH_2_O were used as the negative (0% hemolysis) and the positive control (100% hemolysis), respectively. The solution with red color means the release of hemoglobin from the damaged erythrocytes, and the red pellet at the bottom of the tube is intact erythrocytes formed by centrifugation. **** *p* < 0.0001. PBS, phosphate-buffered saline; ddH_2_O, deionized water.

The biocompatibility of 5FU-CaCO_3_np was evaluated through its cytotoxicity on normal cells, hemocompatibility, and toxicity on brine shrimps. Its cytotoxicity on normal cells was analyzed in NIH3T3 cells. Figure 3A indicates that 5FU-CaCO_3_np led to a gradual increase in the cytotoxicity on NIH3T3 cells in a concentration-dependent manner, with obvious cytotoxic effects at the high concentrations of 120 and 480 μM. Moreover, the IC_50_ value of 5FU-CaCO_3_np was 65.33 ± 11.89 μM. Nonetheless, pure 5-FU showed much stronger cytotoxicity (*p* < 0.05), with an IC_50_ value of 2.12 ± 0.22 μM, which may be attributed to the sustained and prolonged release of 5-FU from 5FU-CaCO_3_np at the pH of the healthy cell environment (pH 7.4). This indicated that 5FU-CaCO_3_np significantly reduced the toxic effects of 5-FU on normal cells.

On the other hand, a strong correlation exists between the outcomes of in vitro hemocompatibility analysis and in vivo toxicity studies, and hemocompatibility analysis serves as a quick in vitro evaluation that can proceed to more resource- and time-intensive in vivo toxicity studies [33]. The hemocompatibility of 5FU-CaCO_3_np was assessed through an erythrocyte-induced hemolysis test which measured the percentage of hemoglobin released upon direct contact of 5FU-CaCO_3_np with erythrocyte surfaces resulting in cell lysis. Figure 3B,C display the results of the hemocompatibility analysis. Hemolysis indicates that the hemolysis ratio is more than 25%, while non-hemolysis is less than 10% [34]. 5FU-CaCO_3_np showed low hemolysis rates (below 12%) even at a high concentration (4000 μg/mL). Based on the above findings, it can be concluded that CaCO_3_np loading 5-FU can reduce the strong toxicity of 5-FU on normal cells, and the synthesized 5FU-CaCO_3_np has favorable hemocompatibility.

The invertebrate brine shrimp has a short life cycle and is well adapted to laboratory conditions, having a low maintenance cost; thus, it was used to analyze the toxicity of 5FU-CaCO_3_np [35,36]. The brine shrimp survival results after treatment of positive control KMnO_4_ suggested that KMnO_4_ had toxicity to brine shrimp. Clarkson’s toxicity index revealed that KMnO_4_ was non-toxic (lethal concentration 50 value more than 1000 μg/mL) within 12 h of exposure but moderately toxic (lethal concentration 50 value between 500 and 100 μg/mL) after further extended exposure of 24, 36, and 48 h [37]. However, 5FU-CaCO_3_np exhibited no toxicity as no dead brine shrimp was recorded in varying concentrations of 5FU-CaCO_3_np even with 48 h of incubation. Overall, 5FU-CaCO_3_np possesses pronounced biocompatibility.

### 3.4. Inhibitory Effects of 5FU-CaCO_3_np Combined with Thymoquinone on CT26 Cell Proliferation

**Figure 4:**
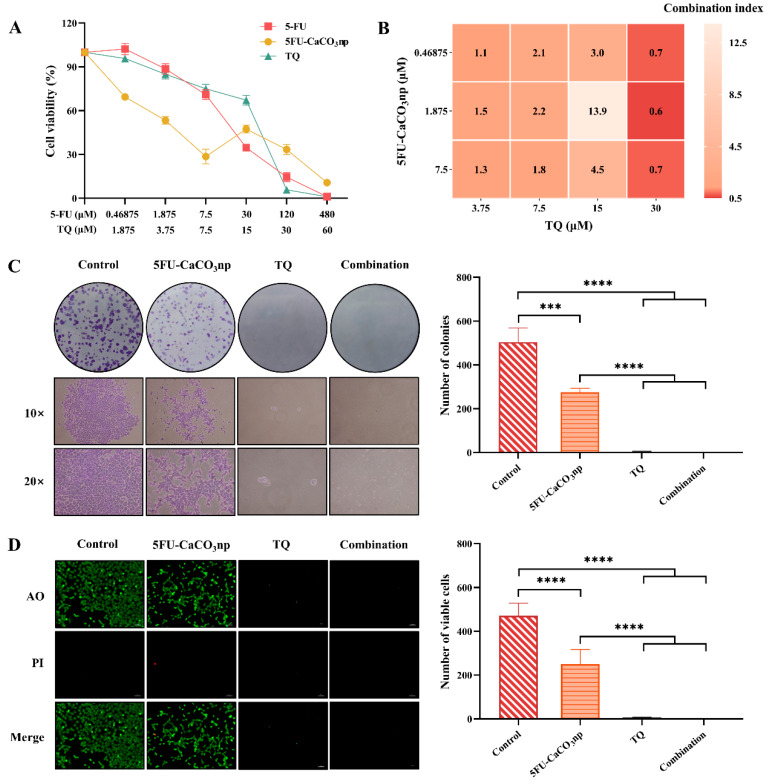
(**A**) Cytotoxicity of 5-FU (5-FU concentrations: 0–480 μM), 5FU-CaCO_3_np (5-FU concentrations: 0–480 μM), and TQ (TQ concentrations: 0–60 μM) on CT26 cells for 96 h; (**B**) combination index heat map of 5FU-CaCO_3_np and TQ in combination against CT26 cells; (**C**) colony formation assay on CT26 cells treated with 5FU-CaCO_3_np (1.875 μM) and TQ (30 μM) alone or in combination, with the represented images under 10× and 20× magnification; (**D**) AO/PI staining assay on CT26 cells treated with 5FU-CaCO_3_np (1.875 μM) and TQ (30 μM) alone or in combination, with the represented images showing viable cells revealed by AO staining in green and non-viable cells revealed by PI staining in red. *** *p* < 0.001, **** *p* < 0.0001.

The combinatorial effects of 5FU-CaCO_3_np and TQ on CT26 cell proliferation were analyzed through MTT, colony formation, and AO/PI staining assays. In vitro cell viability of the synthesized 5FU-CaCO_3_np in CT26 cells was first investigated and compared with the result of pure 5-FU. Figure 4A displays a decreasing trend of cell viability while pure 5-FU concentration increased. Remarkably, 5FU-CaCO_3_np showed a more pronounced reduction, indicating that the delivery system made 5-FU more effective against the cancer cells, with a lower IC_50_ value of 5.06 ± 1.11 μM compared to that (17.23 ± 2.37 μM) of 5-FU. However, at concentrations exceeding 7.5 μM, the decrease in cell viability following treatment with 5FU-CaCO_3_np was gradual, with 5-FU exhibiting a more potent inhibitory effect on cell viability than 5FU-CaCO_3_np. This outcome suggested a sustained and prolonged 5-FU release from 5FU-CaCO_3_np. Therefore, CaCO_3_np was proven to improve the targeted delivery of 5-FU to the colon cancer site. 5FU-CaCO_3_np enhanced the accumulation of 5-FU in colon cancer cells due to its pH sensitivity while minimizing the toxicity of 5-FU to normal cells, leading to higher local 5-FU concentrations and improved therapeutic efficacy. Additionally, 5FU-CaCO_3_np allowed for the controlled release of 5-FU, ensuring a sustained therapeutic effect. Furthermore, it has been reported that 5-FU has poor solubility, which limits its clinical applications [38]. However, compared to 5-FU, the enhanced therapeutic efficacy of 5FU-CaCO_3_np revealed the improved bioavailability of 5-FU by loading into CaCO_3_np.

Inhibition of cell viability of TQ also increased with increasing concentrations, with an IC_50_ value of 13.66 ± 1.60 μM. The combined concentrations of 0.46875, 1.875, and 7.5 μM for 5FU-CaCO_3_np and 3.75, 7.5, 15, and 30 μM for TQ were selected based on their respective IC_50_ values_._ The Chou–Talalay method was used to calculate the CI of 5FU-CaCO_3_np and TQ to analyze their synergistic effects [39]. As displayed in the CI heatmap (Figure 4B), the combination of 5FU-CaCO_3_np (1.875 μM) and TQ (30 μM) resulted in significant inhibition of proliferation in CT26 cells, with the lowest CI value (0.6) observed. Moreover, the colony formation assay and AO/PI staining assay on CT26 cells were conducted to validate the anti-proliferation effects of 5FU-CaCO_3_np, TQ, and the combination of both (Figure 4C,D). Compared to the control group, all treatments inhibited the proliferation of CT26 cells. Notably, the combination of 5FU-CaCO_3_np and TQ exhibited significantly superior inhibitory effects on CT26 cell proliferation than 5FU-CaCO_3_np alone (*p* < 0.0001). These findings demonstrated the superior efficacy of 5FU-CaCO_3_np (1.875 μM) combined with TQ (30 μM) in inhibiting the proliferation of colon cancer CT26 cells.

### 3.5. 5FU-CaCO_3_np Combined with Thymoquinone Induces Apoptosis and Inhibits the Migration of CT26 Cells

**Figure 5:**
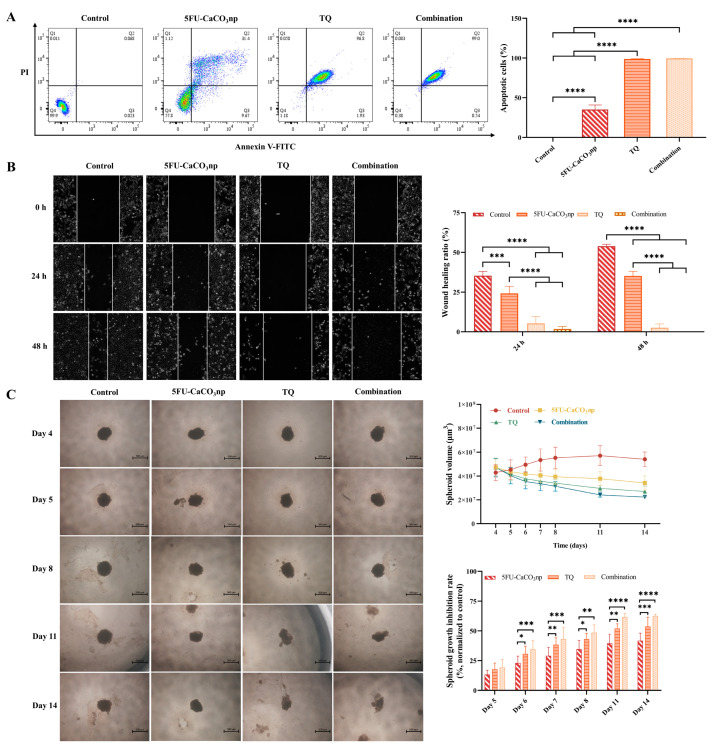
(**A**) Annexin V-FITC assay on CT26 cells treated with 5FU-CaCO_3_np (1.875 μM) and TQ (30 μM) alone or in combination, with the represented images depicting cells in different quadrants: necrosis (Q1), late apoptosis (Q2), early apoptosis (Q3), and live (Q4), where Q2 and Q3 are collectively called apoptotic cells; (**B**) wound healing assay on CT26 cells treated with 5FU-CaCO_3_np (1.875 μM) and TQ (30 μM) alone or in combination, with the represented images showing cell migration after 24 and 48 h; (**C**) growth inhibition analysis of CT26 spheroids treated with 5FU-CaCO_3_np (1.875 μM) and TQ (30 μM) alone or in combination, with the represented images displaying the spheroid volume and growth inhibition. * *p* < 0.05, ** *p* < 0.01, *** *p* < 0.001, **** *p* < 0.0001.

The effects of 5FU-CaCO_3_np, TQ, and their combination on CT26 cell apoptosis were evaluated using flow cytometry. Treatment with 5FU-CaCO_3_np alone at the dose of 1.875 μM induced 41.07% of the cells to become apoptotic in CT26 cells, including 9.67% early apoptotic cells and 31.40% late apoptotic cells (Figure 5A). The addition of TQ (30 μM) remarkably enhanced the 5FU-CaCO_3_np-induced apoptosis, resulting in 99.54% apoptotic cells in CT26 cells, where 99.00% were late apoptotic cells (*p* < 0.0001). This indicated that 5FU-CaCO_3_np in combination with TQ inhibited cell proliferation by inducing cell apoptosis in CT26 cells.

Subsequently, the wound healing assay was used to analyze the cell migration of CT26 cells. Cancer cell migration is a critical process in cancer metastasis which significantly contributes to mortality following the surgical removal of primary tumors [40,41]. Therefore, inhibiting cell migration is a viable strategy for anticancer therapy. As shown in Figure 5B, treatment with 5FU-CaCO_3_np (1.875 μM) or TQ (30 μM) alone inhibited CT26 cell migration. The co-treatment significantly enhanced the inhibition of cell migration by 5FU-CaCO_3_np (*p* < 0.0001). After 24 h of 5FU-CaCO_3_np and TQ co-treatment, the wound healing ratio was 1.78%, indicating a slow cell migration. Comparably, the width of the wound became larger after 48 h with dramatically reduced cell numbers, possibly due to the strong apoptosis induction by 5FU-CaCO_3_np combined with TQ.

### 3.6. 5FU-CaCO_3_np Combined with Thymoquinone Inhibits the Growth of CT26 Spheroids

Compared to 2D cells, 3D spheroids mimic the in vivo environment more closely by allowing cells to interact with each other and the extracellular matrix [42,43]. Therefore, drug responses in 3D spheroids are often more predictive for in vivo responses, making them a better model for preclinical testing. In this study, CT26 spheroids were employed to further investigate the therapeutic effects of 5FU-CaCO_3_np and its combination with TQ. After 4 d of growth, CT26 spheroids were treated with 5FU-CaCO_3_np (1.875 μM) and TQ (30 μM) alone or in combination. As displayed in Figure 5C, the volumes of CT26 spheroids continued to decrease after treatment with 5FU-CaCO_3_np or TQ alone or their combination, and dissociation was observed in response to treatments with prolonged exposure. Previous studies have reported a higher resistance to 5-FU in 3D colon cancer cell spheroids than in 2D cells, with it gradually losing most of its activity in the 3D spheroids [44,45,46]. However, 5FU-CaCO_3_np exhibited increasing spheroid growth inhibitory effects, revealing that 5FU-CaCO_3_np enhanced the sensitivity of CT26 spheroids to 5-FU, thereby improving the therapeutic efficacy of 5-FU on colon cancer cells. This can possibly be attributed to the targeting delivery and sustained release of 5-FU from 5FU-CaCO3np. Notably, disruption of CT26 spheroids was observed on day 11 upon treatment with TQ alone and the combination of 5FU-CaCO_3_np and TQ. Moreover, after normalizing relative to the control group, it was revealed that 5FU-CaCO_3_np combined with TQ exhibited significantly superior spheroid growth inhibition rates to 5FU-CaCO_3_np alone (*p* < 0.01). This confirmed the superior inhibitory efficacy of 5FU-CaCO3np in combination with TQ against cell proliferation.

### 3.7. Molecular Docking

**Figure 6:**
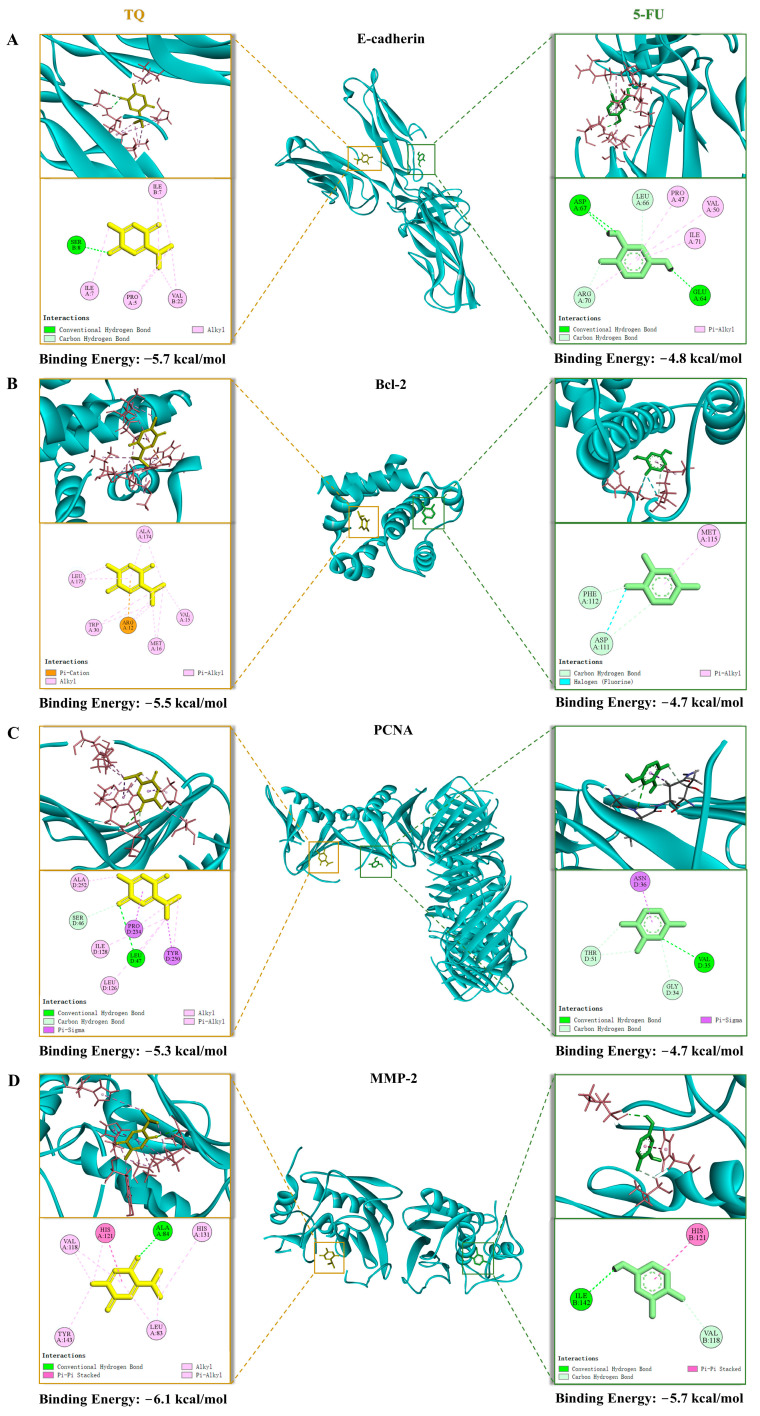
Schematic diagram of molecular docking between 5-FU/TQ and (**A**) E-cadherin, (**B**) Bcl-2, (**C**) PCNA, and (**D**) MMP-2.

To further confirm the effects of 5FU-CaCO_3_np in combination with TQ on proliferation inhibition, apoptosis induction, and migration inhibition in CT26 cells and growth suppression in CT26 spheroids, molecular docking was employed to analyze the interactions between 5FU/TQ and the proteins associated with cell proliferation, apoptosis, migration, and spheroid growth, i.e., E-cadherin, Bcl-2, PCNA, and MMP-2. As a result, the binding energy of 5FU/TQ with E-cadherin, Bcl-2, PCNA, and MMP-2 was less than −4.5 kcal/mol, indicating a significant interaction with these proteins (Figure 6). Interestingly, TQ showed better binding energy than 5-FU as TQ had more interactions with these proteins. 5-FU interacted with E-cadherin, Bcl-2, PCNA, and MMP-2 by forming hydrogen, carbon–hydrogen, pi–alkyl, pi–sigma, and pi–pi stacked bonds. In addition to these bonds, TQ formed alkyl and pi–cation bonds that interacted with the target proteins, where the interactions between the pi electrons of an aromatic ring and a cation were strong, and alkyl interactions contributed to the overall hydrophobic environment in the binding sites, significantly stabilizing the binding of TQ to proteins [47,48]. Additionally, when 5-FU interacted with Bcl-2, a halogen (fluorine) bond was observed at the residue of ASP-111 due to the fluorine atom in 5-FU.

Proliferating cell nuclear antigen (PCNA) is a 36 kDa protein whose expression changes cyclically with DNA replication. It is produced primarily in proliferating and transformed cells and is a specific marker for cell division [49,50]. The B-cell lymphoma-2 (Bcl-2) protein family is a crucial regulator of cell apoptosis and is associated with the development and progression of colon cancer, with Bcl-2 being the representative of anti-apoptotic/pro-survival proteins [51,52]. Additionally, the gelatinase matrix metalloproteinase-2 (MMP-2) is related to angiogenesis and metastasis in colon cancer, whereas E-cadherin promotes cell spheroid formation and is associated with cell metastasis [53,54,55]. Therefore, the favorable interactions between 5-FU and TQ with E-cadherin, Bcl-2, PCNA, and MMP-2 validated that 5FU-CaCO_3_np combined with TQ exhibited anti-colon cancer effects by inhibiting cell proliferation and migration, inducing cell apoptosis, and suppressing spheroid growth.

## 4. Conclusions

In conclusion, the optimal 5FU-CaCO_3_np with pronounced biocompatibility, pH sensitivity, and sustained release properties has been successfully synthesized using a high-speed homogenizer. It is noted that CaCO_3_np loading 5-FU remarkably decreased the cytotoxicity of 5-FU on NIH3T3 normal cells while enhancing its effect against CT26 colon cancer cells. Moreover, 5FU-CaCO_3_np (1.875 μM) and its combination with TQ (30 μM) exhibited therapeutic effects on colon cancer through cell proliferation and migration inhibition, cell apoptosis induction, and spheroid growth suppression, where the efficacy of 5FU-CaCO_3_np combined with TQ against colon cancer significantly surpassed that of 5FU-CaCO_3_np (*p* < 0.05). Molecular docking validated these therapeutic mechanisms by showing the strong interactions between 5-FU/TQ and associated proteins, i.e., E-cadherin, Bcl-2, PCNA, and MMP-2. Given the promising study results of 5FU-CaCO_3_np and its combination with TQ in colon cancer CT26 cells, further study will focus on their efficacy and toxicity in animal models and comprehensive therapeutic mechanism investigation. Overall, 5FU-CaCO_3_np offers an efficacious and low-toxicity potential treatment modality for colon cancer.

## Figures and Tables

**Table 1:** Single-factor experimental design. rpm, revolutions per minute; min, minute.

Factor	Speed (rpm)	Time (min)
Speed	6000	3
	12,000	3
	18,000	3
	24,000	3
Time	6000	1
	6000	3
	6000	5
	6000	7

**Table 2:** FTIR peak assignments of 5FU-CaCO_3_np. FTIR, Fourier transform infrared spectroscopy.

Peak	Peak Assignment	Wavenumber (cm^−1^)
a	In-plane C-O bending	710.68
b	Out of plane C-O bending	851.60
c	Symmetric C-O stretching	1082.21
d	Asymmetric C-O stretching	1440.93
e	Symmetric C-O stretching; in-plane C-O bending	1783.63

**Table 3:** Kinetic analysis of 5-FU release from 5FU-CaCO_3_np at pH 7.4, 6.5, and 5.0 using various kinetic models. R^2^, regression coefficient; n, release exponent.

pH	Zero-Order Model R^2^	First-Order Model R^2^	Higuchi Model R^2^	Hixon–Crowell Model R^2^	Korsmeyer–Peppas Model	
					R^2^	n
7.4	0.84579	0.92832	0.96651	0.86380	0.98258	0.38839
6.5	0.83112	0.90811	0.96204	0.86074	0.98988	0.34495
5.0	0.76977	0.86509	0.92401	0.83058	0.99084	0.27514

## Data Availability

Data will be made available on request.
